# Testicular damage without clinical manifestations in BALB/c mice experimentally infected with Zika virus

**DOI:** 10.1590/1984-3143-AR2023-0124

**Published:** 2024-07-05

**Authors:** Derick Mendes Bandeira, Arthur da Costa Rasinhas, Raphael Leonardo, Marcos Alexandre Nunes da Silva, Eduarda Lima Araujo, Gisela Freitas Trindade, Renata Tourinho Santos, Ygara da Silva Mendes, Ortrud Monika Barth, Debora Ferreira Barreto-Vieira

**Affiliations:** 1 Laboratório de Morfologia e Morfogênese Viral, Instituto Oswaldo Cruz, Fundação Oswaldo Cruz, Rio de Janeiro, RJ, Brasil; 2 Laboratório de Tecnologia Virológica, Instituto de Tecnologia em Imunobiológicos, Fundação Oswaldo Cruz, Rio de Janeiro, RJ, Brasil

**Keywords:** Zika virus, Inbred BALB/c mice, testis

## Abstract

In 2015-2016, the Zika virus (ZIKV) caused a major epidemic in the Americas, increasing cases of microcephaly and Guillain-Barré syndrome. During this period, the discovery of ZIKV sexual transmission intensified studies on the impact of this virus on the reproductive organs. For this study, 2-month-old male BALB/c mice were infected with 1.26 x 10^6^ PFU/mL of ZIKV in solution via the intravenous route. After three, seven, and fourteen days post-infection (DPI), blood and testicle samples were obtained to detect ZIKV RNA. The authors observed that the infected animals had slower weight gain than the control group. Viremia occurred only at 3DPI, and the ZIKV RNA was detected in one testis sample at 7DPI. The histopathological analysis of this organ revealed intense disorganization of the seminiferous tubules' structure, inflammatory infiltrate, necrosis, hemorrhage, fluid accumulation, congestion of blood vessels, and reduced sperm count. Ultrastructural analysis showed nuclear changes in tubule cells, activation of interstitial cells, and morphological changes in spermatozoa, in addition to fragmentation and decreased electron density of the genetic material of these cells. Thus, despite causing predominantly asymptomatic infections, ZIKV can cause significant subclinical and transient damage, including to male reproductive organs.

## Introduction

The ZIKV was discovered in 1947 in Uganda (Dick et al., 1952). This virus has caused few human cases for decades, mainly asymptomatic (Wikan and Smith, 2016), and in 2007 occurred the first major epidemic in the Yap Islands, affecting approximately 73% of the region's residents (Lanciotti et al., 2008). Between 2013 and 2014, a new major epidemic occurred in French Polynesia, with estimates that 11% of the population required hospitalization (Mallet et al., 2015). These data showed that Zika fever already had a brand-new clinical profile. In 2015-2016, a giant epidemic occurred in the Americas, which turned Zika fever into a public health emergency, mainly due to the discovery of the causal association of ZIKV with severe conditions such as microcephaly (Schuler-Faccini et al., 2016), abortion (Martines et al., 2016), and Guillain-Barré syndrome (Araujo et al., 2016). During this period, the release of the virus in semen and vaginal secretion was also discovered, in addition to confirming the ability of the virus to be sexually transmitted (D’Ortenzio et al., 2016).

All these events made research on this pathogen grow exponentially. Using animal models became essential for better comprehending the infection dynamic and developing diagnostic methods, vaccines, and pharmacological treatments. Regarding histopathological studies, the most used models are immunosuppressed mice, followed by immunocompetent mice and non-human primates (Bandeira et al., 2023).

These studies demonstrated the virus has a tropism for different organs (Miner and Diamond, 2017). However, most works focus on the analysis of the brain (Bandeira et al., 2023). Although sexual transmission has aroused the interest of many authors in reproductive organs, data on immunocompetent animals and humans are still scarce. Evidence shows that ZIKV can reduce fertility in male mice (De La Vega et al., 2019). However, the mechanisms behind these phenomena still need to be fully understood.

Thus, the present study aims to evaluate the effects of experimental infection of adult male BALB/c mice about clinical, biochemical, and hematological parameters, viremia, and quantification of viral load in nine different organs, in addition to carrying out a histopathological and ultrastructural study of the testis of infected animals.

## Methods

### Ethical aspects

The Committee on Ethics in the Use of Animals from Oswaldo Cruz Institute approved the request for the use of animals (protocol L-017/2020/A-1). Likewise, the authors performed all experimental infections with a virus sample whose origin was a human case of Zika fever, which occurred in 2015 during the Brazilian epidemic. The Research Ethics Committee of FIOCRUZ allowed the usage of this biological material (CAAE: 59254116.0.1001.5262).

### The animals and study design

For this study, the Institute of Science and Technology in Biomodels (ICTB/FIOCRUZ) provided 39 male BALB/c mice, two months old, weighing between 20 and 25g. The animals were not used previously for any experiment. After their arrival, we randomly separated the animals into ventilated boxes, with a maximum of five mice dividing the same space. All procedures before euthanasia happened at the vivarium of Hélio and Peggy Pereira Pavillion, where the animals were maintained.

The animals were grouped according to the analysis to be performed and subdivided into control or infected mice and by the time of experimental kinetics: three, seven, or fourteen DPI, as shown in Table 1. A previous study of experimental infection of BALB/c mice with ZIKV found that viremia was detectable up to the seventh day of infection (Yu et al., 2017), and other work published shows that testicular damage tends to occur later (12-14 DPI) (Chan et al., 2016). For this reason, we chose 3, 7, and 14DPI as points to evaluate early, intermediate, and late impacts of the infection (from early clinical data to late histopathological evidence of testicular damage). The evolution of body and organ weight and temperature were recorded with three animals from the control group and all 14DPI mice from the microscopy analyses group.

**Table 1:** Division of the 39 BALB/c mice used in this study into groups and subgroups by analyses performed, infection status, and experimental kinetics time.

**GROUP**	**SUBGROUP**			**TOTAL (Animals per group)**
	**Infection status**	**Point of kinetics**	**Animals per analysis**	
Control Group (for clinical analysis and testicular microscopy)^*^	Not infected	3th day	3	9
		7th day	3	
		14th day	3	
qRT-PCR (serum and testis)	Infected	3DPI	5	15
		7DPI	5	
		14DPI	5	
Microscopy analyzes of the testis (BFM and TEM)	Infected	3DPI	5^**^	15
		7DPI	5**	
		14DPI	5**	

**Legend:** DPI (Days Post-Infection). qRT-PCR (Quantitative Reverse Transcription – Polymerase Chain Reaction). BFM (Bright Field Microscopy). TEM (Transmission Electron Microscopy). * These animals were euthanized on the same days as the infected animals, three for each kinetic point, and used as a control for both molecular and microscopy analyses. **All five animals had their testicles analyzed by the BFM technique, and for the TEM analysis, three of them were randomly selected and used.

### Cell, virus origin, cultivation and inoculation

From the previously mentioned human specimen, ZIKV isolation was performed in C6/36 mosquito lineage cells. After inoculating a monolayer of these cells with 100µL of the virus sample, it was incubated for one hour at 28°C for viral adsorption. The cells were maintained in Leibowitz culture medium (L-15), supplemented with 1% non-essential amino acids, 10% tryptose phosphate broth, and 2% fetal bovine serum (Cultilab) in an oven at 28°C. The viral mass titer (stock) was 2.09 x 10^7^ PFU/mL.

In sequence, the diluted solution was subjected to the qRT-PCR technique, which determined that the inoculum load was 1.26 x 10^6^ PFU/mL. The inoculation volume was 100µL through the caudal vein (intravenous route). Uninfected mice were negative control.

### Anesthesia, euthanasia, sample collection, and storage

All animals had the temperature and body weight conferred one hour before euthanasia. In sequence, the mice received a solution of Xylazine (10mg/kg), Ketamine (200mg/kg), and Tramadol (5mg/kg) by the intraperitoneal route. Once sedated, we collected blood samples by cardiac puncture into tubes without anticoagulants.

After euthanasia by cervical dislocation, one experienced professional surgically opened the abdominal cavity of all animals to collect their testis. These organs were weighed on a scale with two decimal places of precision and then stored according to the technique in which they would be processed: electron transmission microscopy (2% glutaraldehyde in cacodylate buffer 0.2M, pH 7.4), bright field microscopy analysis (Millonig’s buffered formalin), and qRT-PCR (freezing at -70°C).

### RNA extraction and viral load quantification

RNA from the original and diluted viral mass was extracted using the QIAamp Viral RNA Mini Kit (250) (QIAGEN). The extraction of RNA in the organ samples was performed with a silica gel column using the IndiSpin® Pathogen Kit (INDICAL Bioscience). In both cases, the procedure followed the protocol provided by the manufacturer entirely.

Taqman Fast Virus 1 Step Kit (Applied Biosystems) was used to amplify the ZIKV genome. The target genomic region was the PrM/E. The sense sequence was 5'-TTG GTC ATG ATA CTG CTG ATT GC-3' (genome position: 835-857); antisense: 5'-CCT TCC ACA AAG TCC CTA TTG C-3' (genome position: 911-890); and the probe: 5'-FAM-CGG CAT ACA GCA TCA GGT GCA TAG GAG-3' (genome position: 860-886).

The qPCR assay was performed in the ABI 7500 (Applied Biosystems, CA). The 20 µL reaction mixture contained 500 nM of each primer and 250 nM of the fluorogenic probe, 5 µL of RNA or plasmid DNA, 5 µL of TaqMan® Fast Virus 1-Step Master Mix (Thermo Fisher Scientific), 7.5 µL of nuclease-free water (Ambion). Amplification conditions were 50 °C for 5 min, 95 °C for 20 s and 40 cycles of 95 °C for 3 s, and 60 °C for 33 s.

### Bright Field Microscopy (BFM) analysis

For BFM analyses, testicles were allocated in histological cassettes, washed in running water for one hour to remove the fixative and allocated in 70% alcohol. Subsequently, the material was embedded in paraffin, sectioned (5µm) for making slides, and stained with hematoxylin and eosin. Images of the main histopathological changes were made from the slides. We also performed quantitative analyses of spermatogenesis and testicular damage. For this purpose, a stained slide was separated from each animal to evaluate 20 seminiferous tubules. Each received a score from one to ten, according to Johnsen's testicular biopsy criteria (Johnsen, 1970). A score of ten is related to the tubule with the most preserved structure, and a score of one represents a tubule with the highest degree of destruction.

### Transmission electron microscopy

The fixated testicle samples were cleaved into fragments of approximately 1mm of edge. After removing the fixative with 0.2M sodium cacodylate buffer in 7% sucrose, the material was post-fixed with 1% osmium tetroxide, dehydrated in increasing concentrations of acetone, and infiltrated with epoxy resin (Barreto-Vieira et al., 2010). The resin was prepared with the EMBed-812 Embedding kit (Electron Microscopy Sciences) according to the protocol provided by the manufacturer.

The blocks were trimmed by shaving the sample area into a trapezoid shape and sectioning it to deposit the ultrathin cuts of 70nm on copper grids. The samples were analyzed using a Hitachi HT7800 transmission electron microscope to assess the ultrastructural changes induced by the infection.

### Statistical analysis

Initially, a Shapiro-Wilk test was performed to determine our data's distribution pattern. Due to the small sample size and non-normal distribution, a comparison of results between groups was performed using the Mann-Whitney test. When a group had n<5 or zero value, the Kuskal-Wallis tests were used. In all cases, the significance level was α=0,05.

The analyses were conducted using the BioEstat 5.0 software (Instituto Mamirauá), and graphs and tables were created using the Excel^®^ software).

## Result

**Figure 1:**
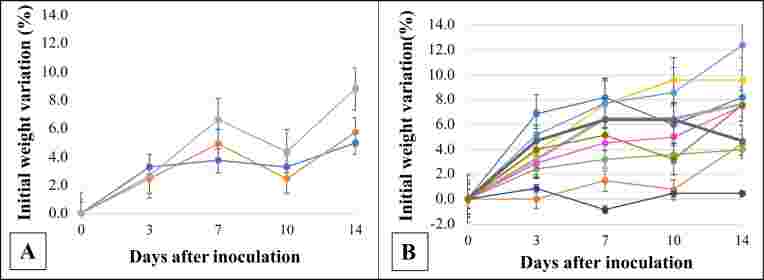
Comparative analysis of weight change in control and infected BALB/c mice along the experimental kinetics. **Legend:** Control BALB/c mice (A). Mice infected with Zika virus (B). The control group represents only animals euthanized at the 14-day point of infection. Each line and color represent a different animal. Statistical comparison of the weight of control and infected animals: 0DPI (*p* = 0.3078); 3DPI (*p* = 0.2296); 7DPI (*p* = 0.2354); 10DPI (*p* = 0.1256); 14DPI (*p* = 0.2361); Kruskal-Wallis test.

No statistically significant changes were observed in the temperature or weight of the animals. However, it is possible to note that infected animals gain weight slower than control mice (Figure 1). There were no deaths nor clinical manifestations associated with viral infection.

**Figure 2:**
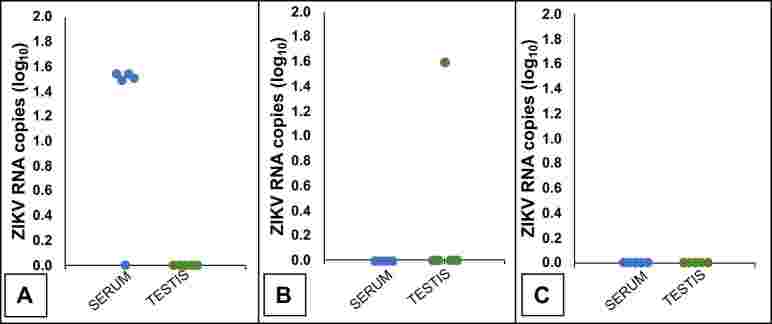
Detection of Zika virus RNA in serum and testis of BALB/c mice experimentally infected with Zika virus. **Legend:** Results obtained at three (A), seven (B), and fourteen days post-infection (C). For proper pairing, animals in the control group were euthanized after three, seven, and fourteen days of follow-up.

**Figure 3:**
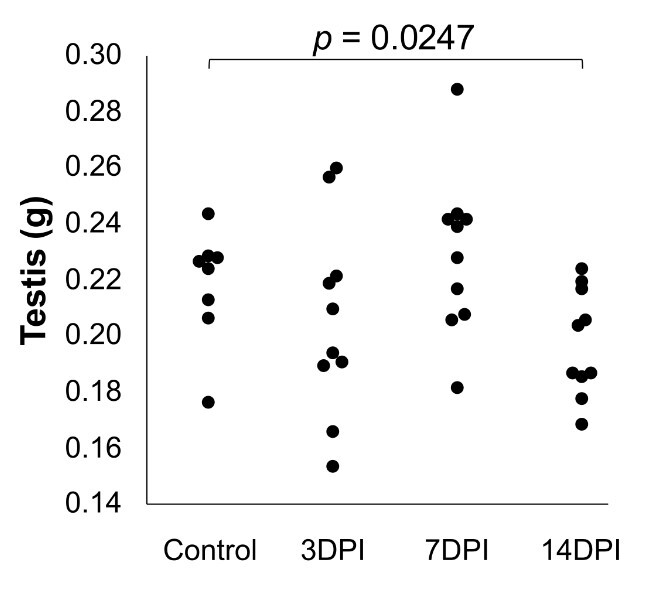
Testicle weight variation in BALB/c mice according to the time of infection. **Legend:** DPI (Days Post-Infection). The weight of the testicles at 3DPI and 7DPI did not show a statistically significant difference to the control group (p > 0.05). Analyzes were performed with the Mann-Whitney test.

Regarding the detection of viral RNA, viremia was detected at 3DPI (four out of five mice), and only one sample of testis had ZIKV RNA detectable (1.6 log_10_ copies at 7DPI) (Figure 2). The mice also showed testicular atrophy, evidenced by a reduction in testicular weight at 14DPI (Figure 3).

**Figure 4:**
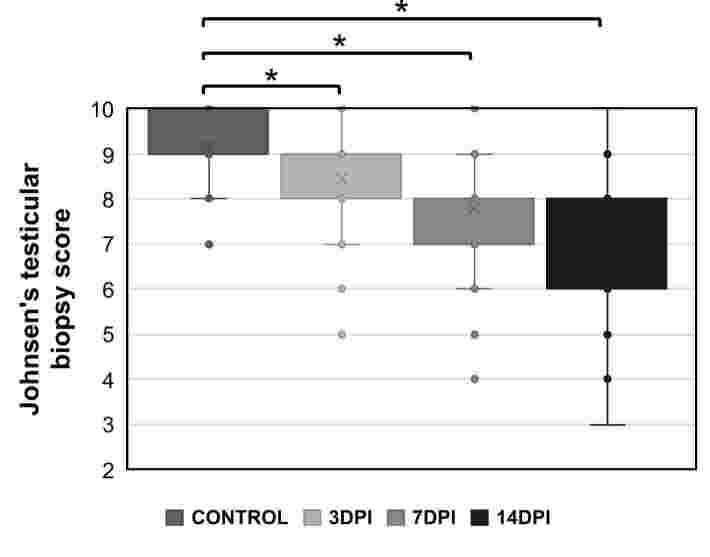
Johnsen’s testicular biopsy score reduction in Zika virus-infected BALB/c mice across experimental kinetics. **Legend:** * p < 0,001. DPI (Days Post-Infection).

Moreover, Figure 4 shows that Johnsen's testicular biopsy score (Johnsen, 1970) gradually decreases with the time of infection, reaching the lowest level also at 14DPI.

**Figure 5:**
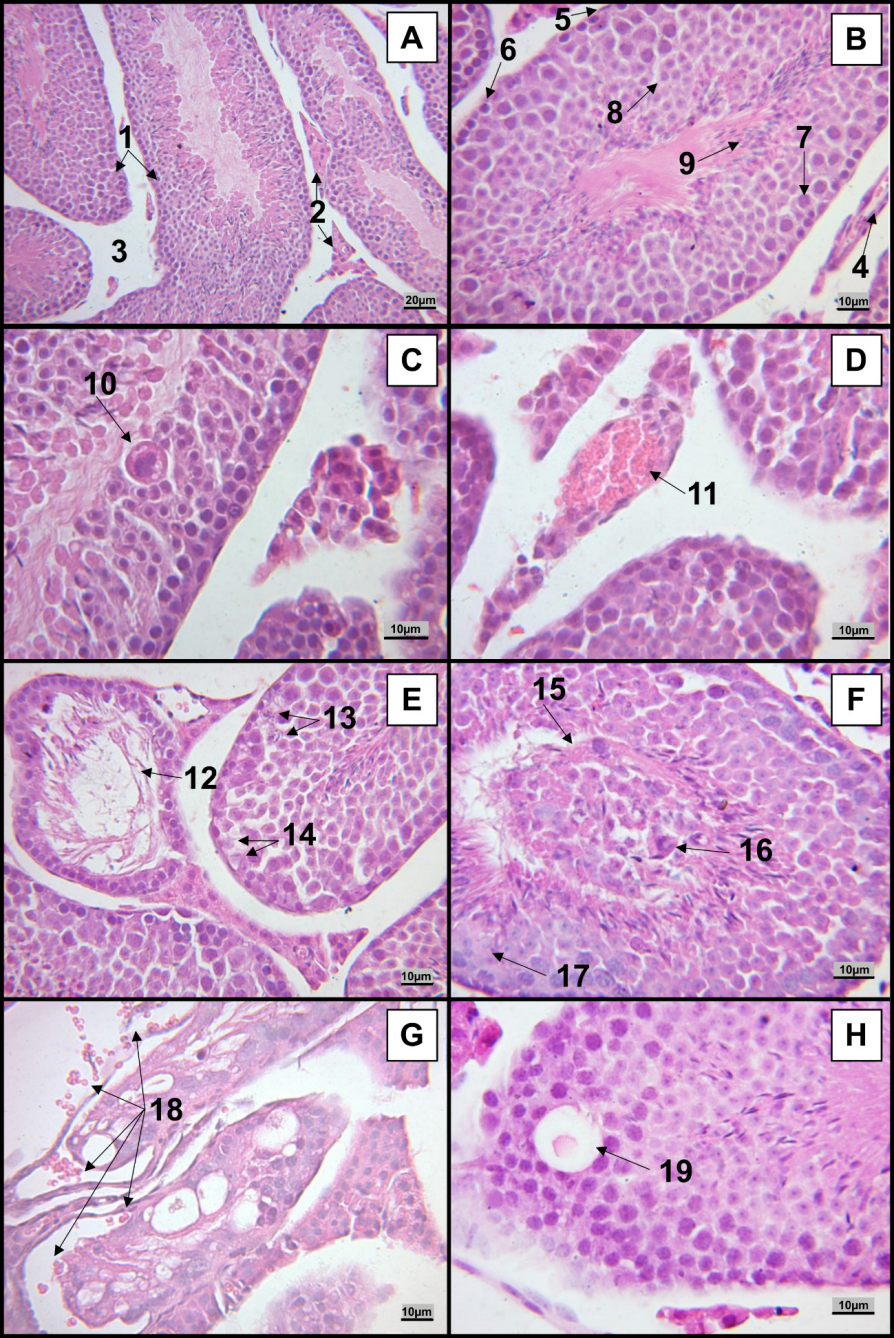
Main histopathological alterations in the testis of BALB/c mice infected with the Zika virus. **Legend**: A – B: control (non-infected) animals; C – H (infected animals). C – F, H: 14DPI mice. G: 7DPI mouse. Typical pattern: A: 1 - Seminiferous tubules; 2 - Interstitial cells; 3 - Interstitial space. B: 4 – Blood vessel; 5 – Myoid cell (squamous cell); 6 – Spermatogonia; 7 - Primary spermatocyte; 8. Spermatids; 9 – Sperm (arrow points to heads; flagella project toward the center of the tubule). Pathological pattern: C: 10. Inflammatory cell with intracellular vacuoles inside the seminiferous tubule. D: 11 – Blood vessel congestion. E: 12 – The seminiferous tubule is almost destroyed, with only the peripheral crown of spermatogonia remaining; 13 – Nuclear atypia; 14 – Ghost cell lesion. F: 15 – Disorganization of the structure of the seminiferous tubule, with cells of the spermatogenic lineage forming an island in the center of the tubule, surrounded by a crown of spermatozoa; 16 – Cell with a horseshoe-shaped nucleus, suggestive of a banded neutrophil; 17 - Cells with weakly stained cytoplasm and nucleus (ghost nucleus). G: 18 – Hemorrhage. H: 19 – Large gap in the seminiferous tubule, filled with liquid.

The histopathological analysis of testis slides (Figure 5) revealed, in infected mice, a wide variety of anomalies in infected mice: inflammatory vacuolated cell inside the seminiferous tubule (Figure5C), blood vessel congestion (Figure 5D), advanced seminiferous tubule destruction (Figure5E), nuclear atypia (Figure 5E), ghost cell lesion (Figure 5E), disorganization of the structure of the seminiferous tubule (Figure 5F), horseshoe-shaped nucleus (Figure 5F), weakly stained cytoplasm and ghost nucleus (Figure 5F), hemorrhage (Figure 5G), and edema inside the seminiferous tubule (Figure 5H). Control mice (Figure 5A-B) did not present these changes in the seminiferous tubules, cell organization, vascular hemostasis, and inflammatory response.

**Figure 6:**
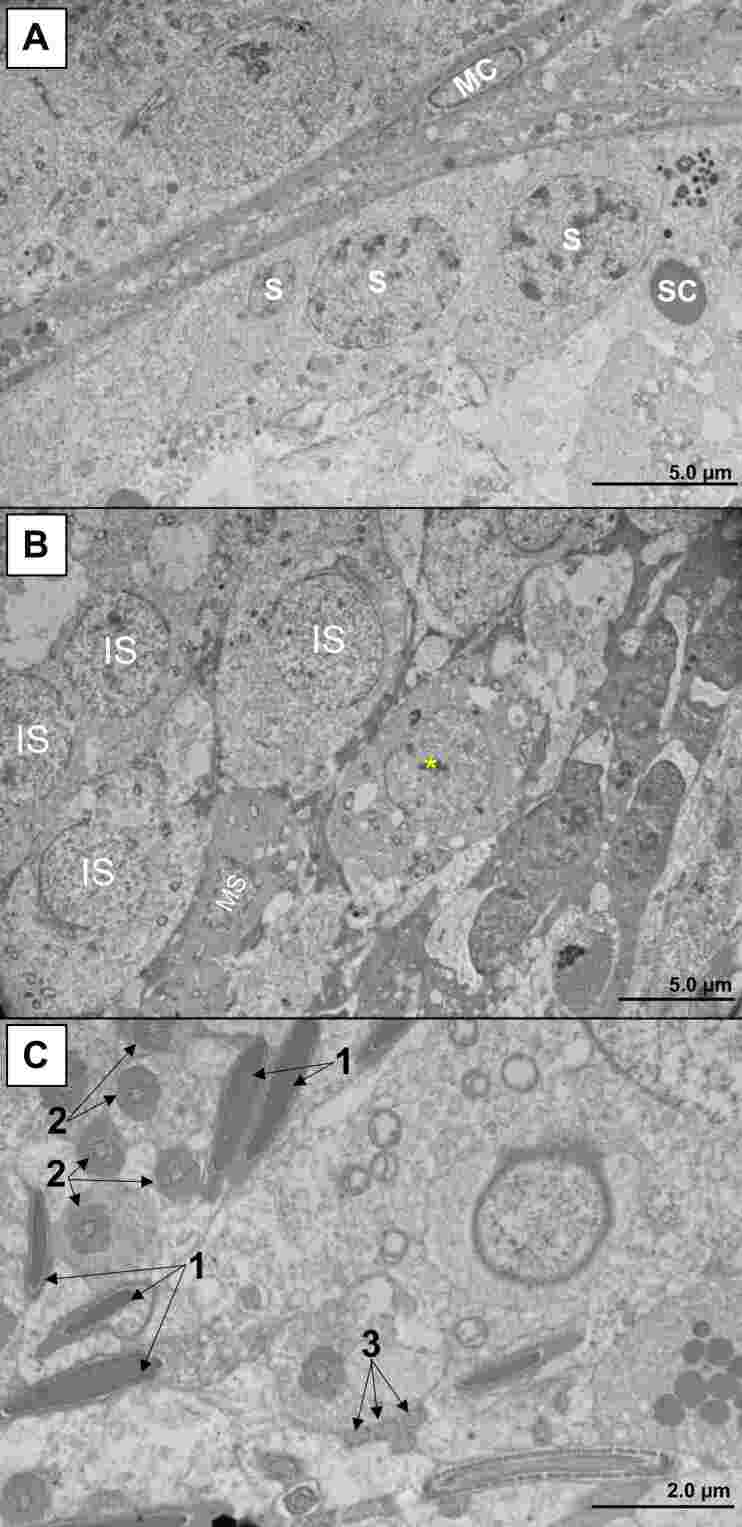
Typical ultrastructural characteristics of testicular tissue in control (not infected) mice, obtained by transmission electron microscopy technique. **Legend:** A. Periphery of the seminiferous tubule. MC (Myoid Cell); S (Spermatogonia); SC (Sertoli cell). B. Intermediate portion of the seminiferous tubule. IS (Immature Spermatid); MS (Mature Spermatid). Asterisk: Spermatid in transition from immature to mature. C. Center of the seminiferous tubule. 1: Head of spermatozoa; 2: Intermediate piece (microtubules surrounded by a crown of mitochondria); 3. Main piece (Flagellum; with fibrous sheath and outer dense fiber). Transmission electron microscopy (Hitachi HT7800 transmission electron microscope).

**Figure 7:**
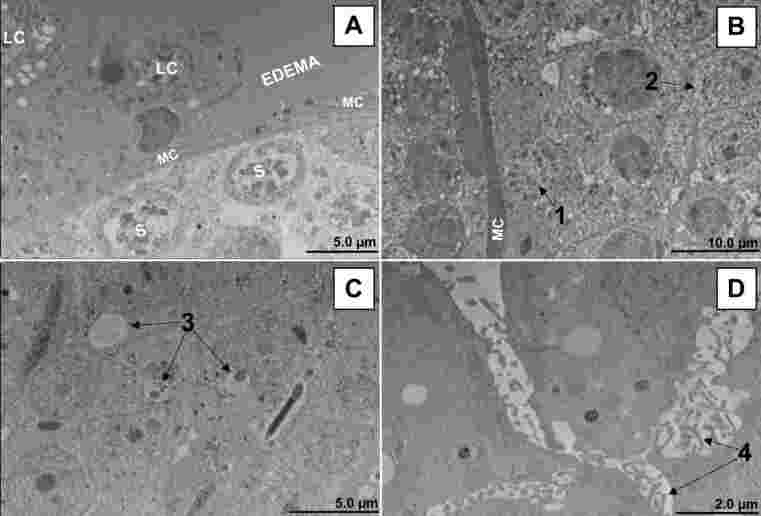
Pathological ultrastructural findings in testis from BALB/c mice infected with the Zika virus (14DPI). **Legend**: A. Periphery of the seminiferous tubule and interstitial space. LC (Leydig cells), MC (Myoid cells), S (spermatogonia). In this region, it is possible to observe the presence of edema in the interstitial space and nuclear pyknosis in the spermatogonia due to the cell death process. B. Seminiferous tubule in which it is possible to observe karyorrhexis in spermatogonia (1) and a spermatid mitochondrial swelling and increased electron density of ribosomes. C. Region closest to the center of the tubule. Note the presence of vacuoles inside the spermatids (3) and low sperm count. D. Interstitial space. Activation of cells is evidenced by the formation of filopodia (4). Transmission electron microscopy (Hitachi HT7800 transmission electron microscope).

**Figure 8:**
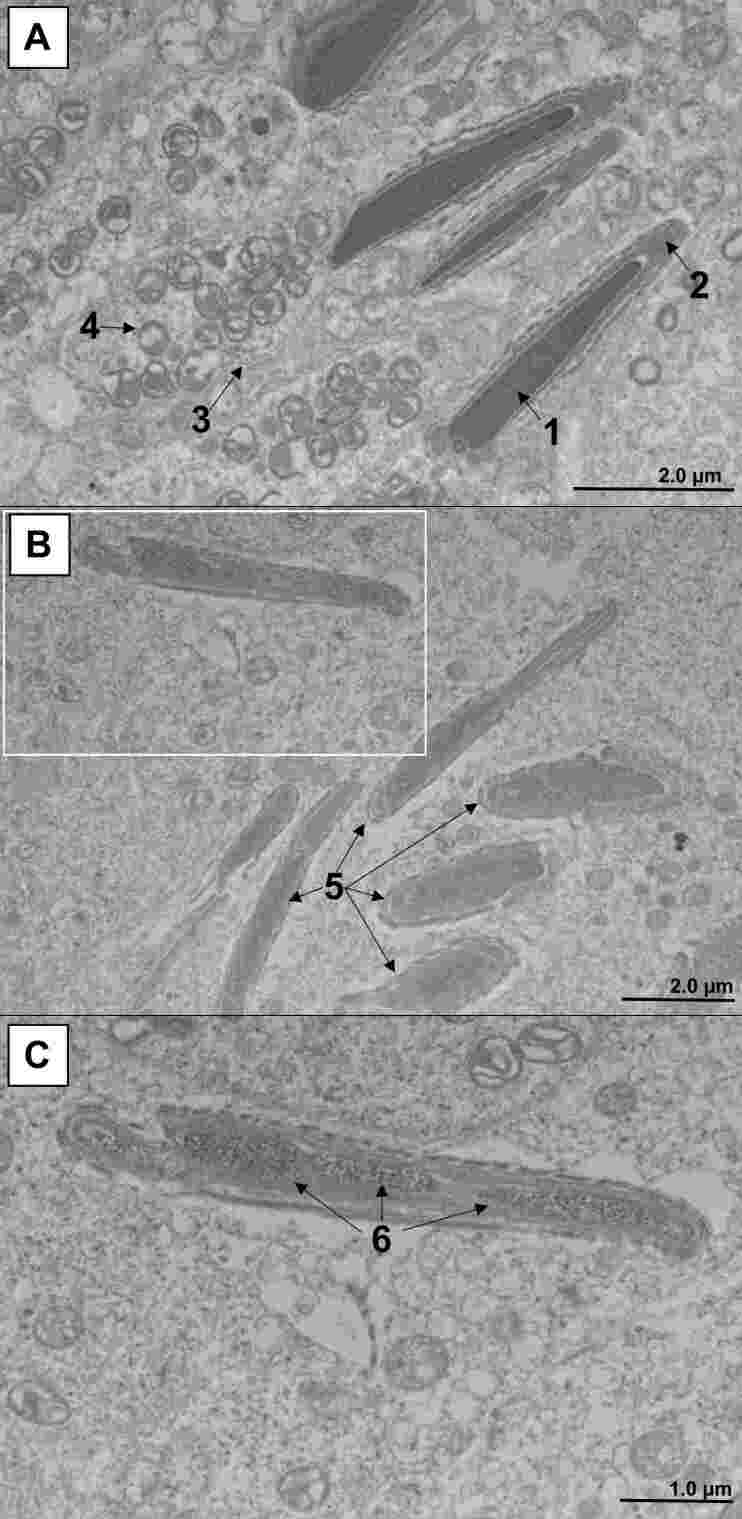
Ultrastructural findings in spermatozoa from BALB/c mice infected with the Zika virus (14DPI). **Legend:** A. Morphology of sperm cells from control (uninfected) animals. Nucleus with electrodense and homogeneous genetic material (1), well-defined acrosome (2), and plasmatic membrane (3), intermediate piece filled with mitochondria (4). B. Sperm morphology in animals infected with ZIKV. Changes in nuclear shape and electron density (5). C. Enlargement of the area highlighted in white in **Figure B.** The sperm's nuclear material is significantly less electrodense and fragmented into three segments (6). Transmission electron microscopy (Hitachi HT7800 transmission electron microscope).

The typical ultrastructural pattern of testis from control mice is shown in Figure 6. In infected animals, we detected relevant abnormalities such as edema (Figure 7A), pyknosis (Figure 7B), karyorrhexis (Figure 7B), mitochondrial swelling (Figure 7B), increased ribosome electron density (Figure 7B), spermatid vacuolation (Figure 7C), and interstitial cell activation (Figure 7D). In addition, there was a considerable reduction in the number of sperm cells in the infected mice. These cells had changes in their morphology, including fragmentation of the genetic material in the nucleus (Figure 8).

## Discussion

Zika virus infection is predominantly asymptomatic in humans (Sharma et al., 2020). Immunocompetent BALB/c mice also reproduce this absence of relevant clinical manifestations. However, our data demonstrate that subclinical lesions occur.

Previous studies reported slower weight gain in mice and non-human primates infected with ZIKV (Hirsch et al., 2018; Chiu et al., 2017). The same pattern happens among human newborns with congenital Zika syndrome (Santa Rita et al., 2017).

Concerning the testicle, the early discovery of the viral tropism for this organ and sexual transmission aroused the scientific community's attention to study this topic (D’Ortenzio et al., 2016). In a recent systematic review our group published, we pointed out seven studies that detected viral load in the testicles of animals and reported histopathological findings that corroborated several data obtained in the experimental infection conducted in this paper (Bandeira et al., 2023). Peregrine et al. (2019) reported macrophage infiltration into the testis of olive baboons. Dowall et al. (2017) also observed this infiltrate and added that sometimes polymorphonuclear cells were also observed. These authors also described a homogeneous and eosinophilic material (possibly a proteinaceous fluid) expanding the interstitium (Dowall et al., 2017). Zhang and colleagues described alterations in the structure of the seminiferous tubules of Tupaia belangeri chinensis (Zhang et al., 2019). Finally, Dowall et al. (2017) reported necrosis of the tubules, and all these data confirm our results.

Another relevant aspect was that our study demonstrated a reduction in testicular weight by 14 DPI. A paper from Clancy and collaborators showed that ZIKV atrophies the entire reproductive system of mice. This atrophy persisted even after 70 days of infection (Clancy et al., 2019). Furthermore, Wang et al. demonstrated that mice infected with ZIKV had reduced sperm count, motility, and morphological changes (Wang et al., 2023), corroborating our data. Moreover, as a final effect of these changes, De la Vega et al. reported that the infection of male mice with ZIKV reduced the fertility of these animals by 44% when the authors placed infected male mice in cages with not infected females (De La Vega et al., 2019).

Finally, it is worth highlighting that, to a certain extent, it is difficult to compare different experimental infection studies in animal models, as the results depend on several factors such as inoculum title, inoculation way, viral strain, sex, age and immunological status of the animal (Bandeira et al., 2023). Intense clinical manifestations and long-term infections tend to be more frequently reported in immunodeficient animals. Our study shows that the immunological competence of BALB/c mice limited viral replication in the testis. However, the inflammatory response generated against ZIKV may be primarily responsible for the damage observed. As an example, it is already known that sperm DNA fragmentation can occur in scenarios of apoptosis and oxidative stress (Muratori et al., 2015), which are expected consequences of the inflammatory response.

To our knowledge, this is the first published work that evaluates the infection and testicular damage caused by ZIKV in an adult, immunocompetent animal model at the histopathological and ultrastructural level. However, the confirmation of the presence of the virus in the tissue by immunohistochemistry and the characterization of inflammatory cells were not financially viable during the experiment period, which are the main limitations of this research. Both procedures are future perspectives of this research to add significant evidence to the histopathological analysis. We also want further to investigate this testicular damage in a more extended cohort and evaluate the impact of ZIKV on semen quality and fertility during the infection and after the recovery.

## Conclusion

ZIKV causes significant damage to the organization of testicular tissue and the structure of sperm cells in BALB/c mice despite their fully responsive immune system. These lesions can be caused either by the direct action of the virus or by the host's inflammatory response to the infection. Although many studies focus attention on ZIKV congenital sequelae in the offspring, this virus also proves to be a dangerous agent of reproductive health reduction for the parental generation. Considering its zoonotic profile and the absence of vaccines and licensed treatment, it is crucial to deepen studies on the impacts of the infection on human and animal reproductive health and strategies to prevent or reduce damage.
